# The Potential Contribution of MyomiRs miR-133a-3p, -133b, and -206 Dysregulation in Cardiovascular Disease Risk

**DOI:** 10.3390/ijms252312772

**Published:** 2024-11-27

**Authors:** Paolina Crocco, Alberto Montesanto, Rossella La Grotta, Ersilia Paparazzo, Luca Soraci, Serena Dato, Giuseppe Passarino, Giuseppina Rose

**Affiliations:** 1 Department of Biology, Ecology and Earth Sciences, University of Calabria, 87036 Rende, Italy; paolina.crocco@unical.it (P.C.); alberto.montesanto@unical.it (A.M.); rossella.lagrotta@unical.it (R.L.G.); ersilia.paparazzo@unical.it (E.P.); serena.dato@unical.it (S.D.); giuseppe.passarino@unical.it (G.P.); 2 Unit of Geriatric Medicine, Italian National Research Center on Aging (INRCA-IRCCS), 87100 Cosenza, Italy; l.soraci@inrca.it

**Keywords:** cardiovascular disease, heart failure, stroke, miRNA-133a, miRNA-133b, miRNA-206

## Abstract

Cardiovascular disease (CVD) is a major global health concern. The number of people with CVD is expected to rise due to aging populations and increasing risk factors such as obesity and diabetes. Identifying new molecular markers is crucial for early diagnosis and treatment. Among these, plasma levels of some miRNAs, specifically expressed in cardiac and skeletal muscle, known as myomiRs, have gained attention for their roles in cardiovascular health. This study analyzed the plasma levels of miR-133a-3p, -133b, and -206 in the pathogenesis of cardiovascular diseases. Using a case–control study design with patients recruited from several nursing homes from Calabria (southern Italy) characterized by different types of CVD compared with non-CVD controls, we found downregulation of miR-133a-3p in heart failure and miR-133b in stroke, along with the overall decreased expression of miR-133b and miR-206 in CVD patients, although they showed low specificity as biomarkers of CVD (as based on ROC analysis). In silico functional characterization of their targets and signaling pathways revealed their involvement in critical cardiovascular processes. Although further research is necessary to fully elucidate their mechanisms and clinical utility, the findings reported here may provide insight into the potential contribution of myomiRs in the cardiovascular injury framework, also offering indications for new research directions.

## 1. Introduction

Cardiovascular disease (CVD), which refers to a wide range of diseases that affect the heart and blood vessels, including ischemic heart disease, stroke, and heart failure, is one of the leading causes of morbidity and mortality worldwide, accounting for more than 17 million deaths each year [1,2]. This condition is highly prevalent in both developed and developing countries, and the rising prevalence of risk factors such as obesity and diabetes, along with population aging, is expected to increase the global burden of CVDs in the coming decades, placing a massive burden on public health worldwide.

The pathogenic mechanisms of CVDs are complex and multifactorial, involving a combination of genetic, environmental, and lifestyle factors, and although recent research has been successful in uncovering novel risk factors and the underlying genetic and molecular mechanisms, seeking novel molecular players involved in the development of CVD is crucial to improve early diagnosis in high-risk patients and develop new potential therapeutic targets.

MicroRNAs (miRNAs) are endogenous, conserved, single-stranded non-coding RNAs of 21–25 nucleotides in length that regulate gene expression post-transcriptionally by base pairing to complementary sequences usually located in the 3′-untranslated region (UTR) of target messenger RNAs (mRNAs), leading to their degradation or translational repression [3]. They are important regulatory elements able to fine-tune almost all biological pathways and are essential for maintaining cellular homoeostasis. Consequently, their dysregulation plays a relevant role in the onset and progression of diverse diseases [4,5].

In the cardiovascular system, miRNAs regulate a wide range of biological processes relevant to cardiovascular health, such as inflammation, oxidative stress, fibrosis, angiogenesis, cardiac cell contractility, and growth [6,7], acting as promoters or suppressors of disease processes. Altered expression of several miRNAs has indeed been associated with several CVDs, holding promise as biomarkers for early disease detection [8,9,10,11,12,13,14,15].

Recently, a small group of miRNAs (referred to as myomiRs) including miRs-1, -133a, -133b, -206, -208a, -208b, -499a, and -499b, highly enriched in skeletal muscle but also expressed in other tissues, including the cardiac muscle, have been shown to have important activities in the functional maintenance of the cardiovascular system as regulators of several processes such as proliferation, differentiation, regeneration, and metabolism [16,17,18,19,20]. Evidence for altered myomiR expression patterns in both human and animal models of cardiovascular diseases has been reported and summarized in recent reviews ([21,22] and references therein). To name a few, decreased expression of both miR-1 and miR-133 was found in mouse and human models of cardiac hypertrophy [23] and reported in patients with myocardial infarction [24]. In the same study by Boštjančič and colleagues [24] miR-208 was found upregulated, in agreement with high expression levels in acute myocardial infarction patients found by Han and colleagues [25]. Likewise, it has been reported that circulating miR-499 was substantially elevated in patients with acute myocardial infarction as compared with healthy control groups [26]. In one report, Kumar and colleagues [27] showed that miR-133b was downregulated in plasma samples of coronary artery disease patients.

Based on the above evidence, we aimed to specifically evaluate the expression levels of this set of muscle-enriched miRNAs (miRNA-1, miRNA-133a-3p, miRNA-133b, miRNA-206, miRNA-208b, and miRNA-499) in patients with different types of CVD and non-CVD controls. The objective was to further highlight the importance of these miRNAs as contributors to CVD pathogenesis and to explore whether they could help discriminate between different subtypes of CVD.

## 2. Results

As stated in the Materials and Methods, subjects were classified as having cardiovascular disease (CVD) when they had one or more of the following physician-confirmed diagnoses: ischemic cardiomyopathy, atrial fibrillation, heart failure, and stroke. The demographic and clinical characteristics of the study cohort stratified according to absence/presence of CVD (CVD−/CVD+) are shown in Table 1. Compared to CVD− controls, CVD+ patients were older (*p* = 0.001), while the ratio of males to females was not significantly different between the two groups (*p*  >  0.05).

Risk factors for CVD, such as diastolic pressure, serum total cholesterol, and low-density lipoprotein (LDL) cholesterol levels, were significantly lower in CVD patients than in control subjects (*p* < 0.01). This is probably due to patients’ use of pharmacological treatments to manage blood pressure and lipid levels. Conversely, levels of uric acid, an emerging biomarker of CVD, as well as markers of kidney dysfunction (urea and creatinine), were higher in CVD patients compared to controls. Finally, as expected, patients with CVD had a higher mortality rate during the study follow-up (49% vs. 20%).

qRT-PCR was conducted to quantify the levels of miR-1, miR-133a-3p, miR-133b, miR-206, miR-208b, and miR-499 in the plasma of healthy controls and CVD patients. For data analysis, we eliminated miR-1, miR-208, and miR-499 since the expression levels were below the threshold of detection to be quantified reliably and analyzed for differential expression.

First, we explored the association between these miRNAs and the variables in Table 1 in the overall study population. A slightly positive correlation between miR-133a-3p and age (r  =  0.14, *p*  =  0.042) and a very weak negative correlation with the level of albumin and HDL cholesterol (r  =  −0.15, *p*  =  0.037 and r  =  −0.17, *p*  =  0.02, respectively) were observed for miR-133a-3p. No other significant correlations were observed.

**Figure 1:**
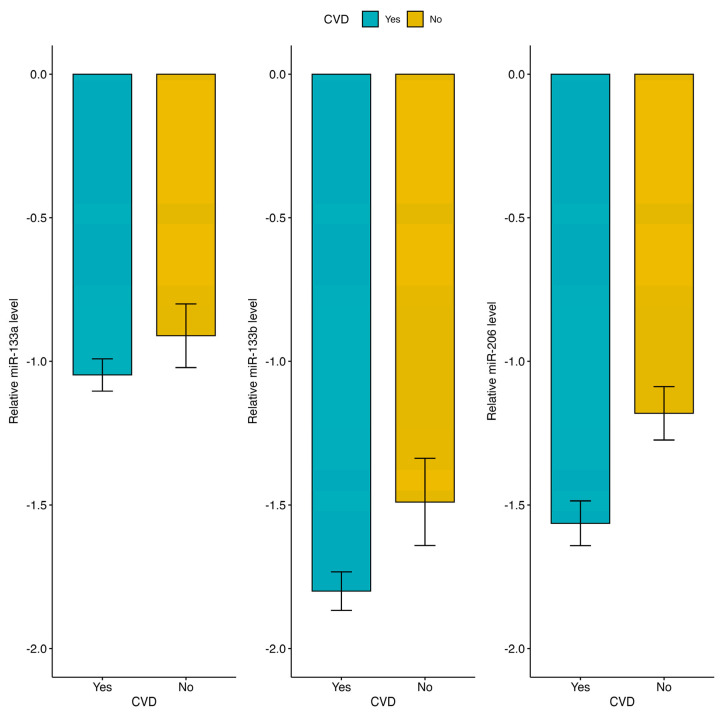
Relative expression of mirR-133a-3p, -133b, and -206 in plasma from CVD and non-CVD subjects. Data are reported as log 2^−∆Ct^ normalized to U6 expression.

We then examined whether the levels of circulating miR-133a-3p, miR-133b, and miR-206 were associated with CVD diagnosis. As shown in Figure 1, the expression levels of miR-133b and miR-206 were significantly lower in CVD samples than in control subjects (*p* < 0.05).

Logistic regression analysis adjusted for age, sex, and statin exposure (Table 2) confirmed this result, showing that a unitary increase in miR-133b and miR-206 expression was associated with a reduction in CVD risk (OR = 0.66; 95% CI = 0.44–0.97; *p* = 0.038 and OR = 0.57; 95% CI = 0.36–0.85; *p* = 0.009 for miR-133b and miR-206, respectively).

Next, to assess the relationship between the expression levels of these miRNAs in the two sample groups, Spearman correlation analysis was employed. The results, depicted in Figure S1, indicated a significant positive correlation between miR-133b and miR-206 expression levels in the CVD group (r = 0.341; *p* < 0.001), suggesting a potential co-regulation of these miRNAs in the context of cardiovascular disease. In contrast, no significant correlation was observed in the control group (r = 0.076; *p* > 0.05).

ROC curve analysis was then used to assess the diagnostic ability of the two miRNAs in discriminating patients with CVD from those without CVD. It was found that neither miR-133b (AUC = 0.596; 95% CI: 0.496–0.696) nor miR-206 (AUC = 0.602; 95% CI: 0.520–0.684), as well as their combination, efficiently discriminated between the two groups.

Since all subjects were followed for a median duration of about three years for their overall survival (28 healthy controls (27%) and 40 CVD patients (49%) died during this period), we also evaluated whether the detected effects of the analyzed miRNAs on CVD risk could affect the subjects’ survival. The miRNA levels were divided by quartiles, and the highest quartile was compared with the others. Kaplan–Meier survival curves were plotted and are shown in Figure S2. For all miRNAs analyzed, *p*-values for both a log-rank test and Cox proportional hazards regression analysis adjusted for age and sex indicated no statistically significant differences in death from all causes between “high-expression” and “low/medium-expression” groups.

Next, we performed a subanalysis to evaluate whether a characteristic miRNA expression profile is related to a specific cardiovascular condition. The results, reported in Table 3, indicated that, compared with the control group, the unitary increase in plasma levels of miR-133a was significantly associated a 53% reduction in the odds of heart failure (OR = 0.47, C.I. 0.24–0.95; *p* = 0.031); a similar trend, although not significant at a nominal level, was observed for miR-206 (OR = 0.62, C.I. 0.37–1.06; *p* = 0.074). In addition, increased serum levels of miR-133b were significantly associated with an approximately 37% reduction in stroke risk (OR = 0.63, C.I. 0.39–0.99; *p* = 0.048).

### In Silico Functional Characterization

**Figure 2:**
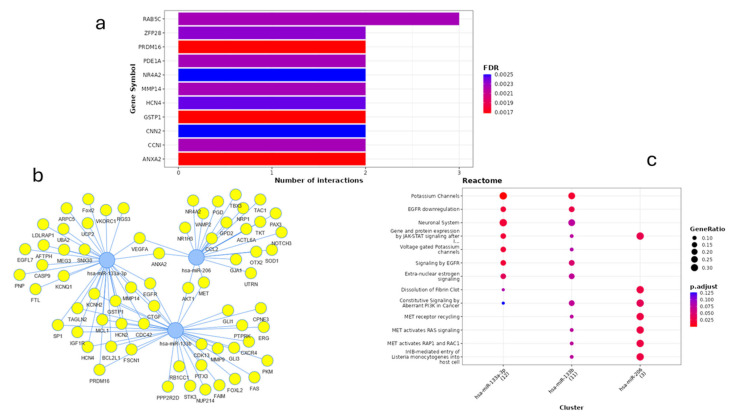
miRNA–target enrichment analysis by MIENTURNET. (**a**) Bar plot of the results of the enrichment analysis obtained as obtained from miRTarBASE. On the Y-axis are the top-ranked target genes of the submitted miRNAs, while the X-axis represents the number of miRNAs targeting them. The color code reflects the adjusted *p*-values. The threshold for the minimum number of miRNA–target interactions was set up to 1; (**b**) Graphical representation of the network of miRNA–target interactions identified by the enrichment analysis; (**c**) Functional enrichments for miRNA target genes according to REACTOME. Dots are colored by *p*-adjusted value (FDR) and sized by gene ratio per pathway (i.e., the number of miRNA targets found enriched in each category over the number of total genes associated with that category).

We performed miRNA–target enrichment analysis by using the MIENTURNET tool (http://userver.bio.uniroma1.it/apps/mienturnet/, accessed on 2 September 2024), setting the three miRNAs as the inputs and using experimentally validated miRNA–target interactions (miRTarBase). We retrieved one hundred and thirty-nine genes significantly associated with these miRNA markers, see Supplementary Table S1. As reported in this table, fifty-seven mRNAs were predicted to be targeted by miR-133a, forty-seven by miR-133b, and thirty-five by miR-206. Fifty-six mRNAs were shared between miR-133a and miR-133b, while four targets were shared between miR-133a and miR-206 and two between miR-133b and miR-206. Three mRNAs were targeted by all three miRNAs. Figure 2a shows the top-ranked targets of these miRNAs and the number of miRNA interactions for each target, while Figure 2b shows the network of miRNA–target interactions identified by the analysis. Functional enrichment analysis for miRNA target genes using the REACTOME database revealed several significantly enriched pathways (Figure 2c). Among these pathways, target genes of miR-133a and miR133b were involved in potassium channels, EGFR signaling, and JAK/STAT signaling pathways. The latter pathway was also targeted by miR-206; other targets of this miRNA are involved in the dissolution of fibrin clots and mainly in signaling by the receptor tyrosine kinase MET.

## 3. Discussion

In recent years, interest has grown in determining the role of miRNAs in the onset and progression of CVD, a leading cause of worldwide morbidity and mortality.

In this study, we focused on myomiRs, a class of miRNAs highly enriched in skeletal and cardiac muscle, which have recently emerged as key players in a range of CVD-related processes, such as hypertrophy, fibrosis, and inflammation (see references in Section 1).

Three of the six selected myomiRs, miR-133a-3p, miR-133b, and miR-206, were detectable in plasma samples and were further analyzed. We observed significantly lower levels of miR-133b and miR-206 in patients with CVD, including ischemic cardiomyopathy, atrial fibrillation, heart failure, or stroke, compared with healthy controls. Moreover, the significant positive correlation between miR-133b and miR-206 in the CVD group suggests a potential cooperative role in the pathogenesis of cardiovascular disease. This contrasts with the absence of correlation in the control group, indicating that these miRNAs may become more interconnected under conditions of cardiovascular injury. Examining specific types of CVD, we found significant downregulation of miR-133b in stroke patients and significant downregulation of miR-133a-3p in heart failure patients, with a trend in the same direction for miR-206.

Taken together, these findings suggest that specific molecular targets and cellular mechanisms involved in the action of these miRNAs are likely differentially activated in response to various cardiac insults, leading to distinct expression patterns in different types of CVD. This view is supported by the results of several studies on various cardiovascular conditions, which highlight the complex and context-dependent roles of these miRNAs in CVD development.

In line with our findings, a study by Yu and co-workers in 2020 [28] found miR-133b downregulated in 60 stroke patients compared with 60 healthy controls.

Two studies, which analyzed miRNA dysregulation in intracranial aneurysm tissue samples, reported a downregulation of miR-133b expression in aneurysmal tissue compared with control vessels from matched patients [29,30]. Consistently, it has been shown that the overexpression of miR-133b enhances functional recovery after stroke in rats [31] and that miR-133b-containing exosomes improve neuronal function, survival, and reduced brain injury volume in a rat model of intracerebral hemorrhage [32]. Decreased expression of this miRNA has also been linked to ventricular fibrillation in patients with MI [33] and observed in patients with coronary artery disease (CAD) compared with healthy controls [27]. However, some contrasting studies have also been published, showing upregulation of miR-133b in patients with ST-segment elevation MI and in a mouse model of myocardial infarction [34]; additionally, higher plasma miR-133b levels have been associated with lipid-rich coronary plaques in patients with stable CAD [35].

As for miR-133a-3p, in line with our finding of downregulation of this miRNA in patients with heart failure, a recent meta-analysis on miRNA expression profiling in heart failure [36] reported that miR-133a, among others, is downregulated in heart failure. Additionally, Sang et al. [37] reported downregulation of miR-133a in cardiac tissue obtained from patients with chronic heart failure and in rat models of the disease. The same study demonstrated that increasing miR-133a expression, either by mimic administration or overexpression, significantly reduced heart fibrosis in rats with chronic heart failure. Consistently, synthetic miR-133a has been shown to prevent heart failure in a pressure overload model [38]. However, mixed results have also been shown, in which increased expression of miR-133a has been identified in heart failure patients compared with controls [39,40]. It is noteworthy that, in CVD, the expression of miR-133a, one of the most abundant cardiac-specific myomiRNAs, can be upregulated or downregulated due to pathological alterations in specific cell types or specific CVD conditions. For instance, while it is downregulated in patients with myocardial infarction (MI), its expression level is significantly elevated in patients with acute myocardial infarction (AMI), for a review, see ref. [41]. Moreover, a very recent study found that serum exosomes induced by ischemic preconditioning (IPC-exo) mitigate excessive replacement fibrosis, improving cardiac function in rats following MI by transferring miR-133a-3p [42]. On the other hand, results of a meta-analysis showed that circulating levels of miR-133a-3p were significantly upregulated in a subgroup of patients with premature coronary heart disease [43]; similarly, upregulation in CAD has been reported [44]. In contrast, Abdallah et al. (2022) reported instead that this miRNA was downregulated in CAD patients [45]. Additionally, the study by Maitrias et al. (2015) found higher levels of miR-133a in symptomatic carotid plaques, linked to advanced atherosclerosis and increased stroke risk, compared to asymptomatic plaques [46]. We did not find an association between miR-133a and stroke, and this difference may be due to variations in atherosclerosis stages or tissue types, as miR-133a could be more prevalent in plaques at certain disease stages.

Concerning miR-206, this myomiR is specifically and highly expressed in skeletal muscle, but only barely detectable in the heart. Yet, a recent study [47] found it differentially expressed in the pericardial fluid of patients with arrhythmic right ventricular cardiomyopathy. Its differential expression in various cardiac conditions has been reported in several studies, mostly in model organisms. For instance, it has been demonstrated that miR-206 is significantly downregulated in rat myocardial tissue following ischemia–reperfusion injury and that its inhibition ameliorates ischemia–reperfusion in arrhythmia models [48,49]. In rat hearts with acute MI, miR-206 expression is reduced in infarcted areas and hypoxia-induced cardiomyocytes, and loss-of-function of miR-206 increases cardiomyocyte apoptosis [50]. Conversely, Shan et al. [51] reported increased expression of miR-206 in a rat model of MI. Additionally, studies have linked miR-206 upregulation to cardiac hypertrophy [52] and arrhythmia in adult cardiomyocytes [53]. In humans, Zhou et al. (2016) found miR-206 significantly upregulated in blood of patients with CAD compared to healthy individuals [54], while Xing et al. (2017) found this miRNA downregulated in atherosclerosis tissue samples, also finding that upregulation of miR-206 in vascular smooth muscle cells (VSMCs) reduced cell survival rate [55]. In our study, miR-206 was found to be downregulated in the entire group of patients with CVD, with a slight decrease observed in patients with heart failure. Although not statistically significant, this finding aligns with the potential role of miR-206 in the regulation of heart muscle growth, which may contribute to heart failure [52,56]. This finding is consistent with a recent review and meta-analysis that studied the diagnostic potential of circulating miRNAs to detect different forms of chronic heart failure, identifying miR-206, among others, as a potential diagnostic biomarker for heart failure with preserved ejection fraction [57].

The discrepancy in results across the studies could be due to a variety of factors, including methodical differences such as the source of samples and the analytical procedures, different patient ethnicities, and sample sizes. On the other hand, the differential expression patterns of miRs-133a, -133b, and -206 may likely depend on the subtype of CVD and the clinical and physiological context in which they exert their function. This raises the possibility that they may serve as diagnostic and/or prognostic markers not only for CVD in general but also for specific CVD types, pathophysiological processes, and/or clinical outcomes. This possibility only comes through a better understanding of the regulation of these miRNAs, as well as elucidation of their relevant targets, their downstream pathways, and their biological functions in the context of the cardiovascular system, which currently have not been fully clarified.

miR-133a-3p forms a cluster with miR-1-2 on chromosome 18 (18q11.2), which is transcribed as a bicistronic transcript regulated by many myocytes’ differentiation factors, including myocardin, a nuclear protein that promotes the activation of cardiac gene expression by associating with the transcription factor serum response factor (SRF) [58]. miR-133b and miR-206 are clustered on chromosome 6 (6p12.2) and are transcribed as a single bicistronic lncRNA or, under certain conditions, by separate promoters to yield separate primary transcripts [59]. The primary transcript encoding miR-133b/miR-206 is induced by Wnt/β-catenin signals [60]. This signaling pathway regulates the stability and nuclear localization of β-catenin and plays an important role in cardiac function and cardiac tissue homeostasis [61,62]. Its activation correlates to pathological stages following MI, including inflammation, angiogenesis, and fibrosis [63,64].

To better highlight the role of the analyzed miRNAs in CVD risk, we conducted a bioinformatics analysis to identify the target genes and pathways regulated by these miRNAs. Target enrichment analysis, based on experimentally validated miRNA–target interactions, identified eleven genes regulated by more than one of the three miRNAs, many of which are implicated in cardiac function and disease. Both miR-133a and miR-133b target PRDM16, a transcription factor essential for normal heart function. Deletion of PRDM16 leads to cardiac hypertrophy, fibrosis, mitochondrial dysfunction, and heart failure [65]. These miRNAs also target the glutathione transferase P1 (GSTP1), an enzyme involved in detoxification and a potential biomarker of myocardial stress and heart failure [66,67], and the hyperpolarization-activated cyclic nucleotide-gated 4 (HCN4) channel, a transmembrane protein which regulates heart rate [68]. Notably, reduced levels of miR-133a lead to increased HCN4 expression in hypertrophic heart [69]. miR-133a and miR-206 target both Annexin A2 (AnxA2) and the nuclear receptor NR4A2. AnxA2 is a calcium-binding protein crucial for heart health, implicated in various cardiovascular processes like angiogenesis, thrombosis, and lipid metabolism [70,71]. NR4A2, on the other hand, has been linked to impaired cardiac function and heart failure [72,73]. Functional enrichment analysis revealed that validated targets of both miR-133a and miR-133b are linked to biological processes that regulate cardiovascular function. These include potassium channels and transmembrane proteins that control cardiac repolarization and the shape and duration of the cardiac action potential and are implicated in various cardiac diseases [74,75,76]. Additionally, the analysis highlighted processes mediated by the EGFR signaling pathway, a key signaling hub linked to vascular physiology and cardiovascular health and disease [77,78,79].

The target genes of all three miRNAs were enriched in the Janus kinase/signal transducer and activator of transcription (JAK/STAT) pathway. This cytokine-regulated pathway, involved in development, homeostasis, and inflammation, plays a crucial role in transmitting stress and growth signals in various myocardial injuries, including apoptosis, hypertrophy, fibrosis, and ischemia [63,80,81]. A recent study by Dong et al. [82] reports that miR-206 protected cardiomyocytes from lipopolysaccharide (LPS)-induced inflammatory injury by inhibiting the JAK2/STAT3 signaling pathway.

miR-206 targets were enriched in gene sets related to the MET signaling pathway, which is activated by the hepatocyte growth factor (HGF) receptor. MET signaling regulates heart homeostasis, prevents oxidative stress, and plays a cardioprotective role in heart injuries such as ischemia by promoting cell survival and inhibiting apoptosis and autophagy [83,84]. Additionally, an enriched pathway was associated with fibrin clot dissolution, the final step of coagulation. Impaired properties of fibrin clots are characteristic of patients with acute and chronic CVDs [85].

While this study contributes to the growing body of evidence underscoring the dysregulation of myomiRs in the framework of cardiovascular injury, it is important to acknowledge its limitations. First, the small sample size of the whole cohort and subgroups of patients with different types of CVD may have affected the reliability of the ROC curve and limit the generalizability of study findings; second, the study cohort consisted of older nursing home residents enrolled in the Calabria region of southern Italy, and although this is relevant to the study of CVD in the elderly population, the results may not be applicable to other age groups and geographic populations, as genetic and environmental factors may influence myomiR expression.

Additional studies, comprising functional validation experiments and a larger cohort of individuals, may provide a more comprehensive and definitive assessment of the potential clinical utility of myomiRs as biomarkers for CVD.

## 4. Materials and Methods

### 4.1. Study Population

The cohort of this retrospective study included 209 individuals, comprising 90 patients (31% were males) with a clinical diagnosis of CVD that included ischemic cardiomyopathy (52%), atrial fibrillation (29%), heart failure (18%), and stroke (31%) and a control group of 119 healthy controls (28% were males). The mean age of the study population was 85 ± 6.3 years for patients with CVD and 82 ± 7.8 years for the healthy controls.

All participants were recruited from nursing homes located in the provinces of Crotone and Cosenza, Calabria region (southern Italy), as part of a larger study aimed at assessing and monitoring the quality of aging in the entire region. Subjects were eligible to participate in the study if they were of Calabrian ancestry.

### 4.2. Clinical and Laboratory Assessments

Each participant underwent a complete clinical and geriatric assessment designed to evaluate cognitive status, functional abilities, physical health, and social aspects. A structured questionnaire administered by a trained interviewer was used to collect data. Demographic (age, sex) and anthropometric (height, weight, BMI) variables were available for all participants. Clinical variables included systolic and diastolic blood pressure (SBP and DBP, respectively), history of hypertension and diabetes, and presence of CVD. The diagnosis of CVD was based on patients’ detailed medical histories, clinical symptoms, imaging studies, and physical or laboratory findings as determined by a board-certified cardiologist in accordance with the international clinical guidelines. Additionally, a peripheral blood sample was drawn from each participant for laboratory examinations, including traditional biomarkers and myomiR, assessed via standardized protocols. Traditional biomarkers included fasting lipid levels (total cholesterol, triglycerides, and high- and low-density lipoprotein cholesterol (HDL-c and LDL-c, respectively), fasting plasma glucose, HbA1c, urea, creatinine, electrolytes, uric acid, total proteins, albumin, C-reactive protein (CRP), iron, ferritin, and total bilirubin.

### 4.3. Plasma RNA Extraction

The peripheral blood specimens from all participants were collected in an EDTA-containing tube and centrifuged at 12,000 rpm for 15 min. Afterward, the plasma was stored in a freezer at −80 °C until analysis. miRNAs were extracted from 200 µL of plasma using the MiRNeasy Serum/Plasma miRNA extraction kit (Qiagen, Hamburg, Germany), following the manufacturer’s protocol. In brief, QIAzol lysis buffer (1000 µL) was added to the sample to facilitate lysis. Upon addition of 200 µL of chloroform and 3.5 μL of Arabidopsis thaliana miR-159a (assay ID 000338), serving as a spike-in control to monitor any inter-sample discrepancies in extraction efficiency and subsequent reverse transcription, the lysate was separated into organic and aqueous phases by centrifugation, with RNA partitioned into the upper aqueous phase which was separated and mixed with 1.5 volumes of 100% ethanol and purified on the RNeasy MinElute spin column provided in the kit. RNA was eluted in 14 µL of RNase-free water. RNA purity was checked using the NanoDrop ND-1000 spectrophotometer (Thermo Fisher Scientific, Milan, Italy) assessing the A260/A280 A260/A230 ratios, which were found to be higher than 1.9. The RNA was quantified on a Qubit 2.0 fluorometer (Life Technologies, Milan, Italy). The yield was around 30–50 ng/mL for each sample.

### 4.4. Quantitative Real-Time Polymerase Chain Reaction

First, 5 μL of RNA was reverse transcribed to cDNA using a TaqMan^®^ microRNA Reverse Transcription Kit (Life Technologies) and stem–loop-specific RT primers for each of the miRNAs examined: hsa-miR-1 (ID 002222); hsa-miR-133a (ID 002246); hsa-miR133b (ID 002247); hsa-miR-206 (ID 000510); hsa-miR-208b (ID 002290); hsa-miR-499 (ID 001045). RNU6B (U6) (ID 001973) was used as an endogenous control to normalize extracted endogenous miRNA levels. The reliability of RNU6B as endogenous was verified by comparing Ct values of U6 between non-CVD and CVD patients. No significant difference was considered for *p* > 0.05.

After incubation, quantitative real-time PCRs were performed using the TaqMan MicroRNA Assay Kit (Applied Biosystems, Milan, Italy). Each PCR contained 1.33 μL of the RT reaction product, 10 μL of TaqMan 2×Universal PCR Master Mix without uracil-N-glycoslyase (UNG), and 1 μL of 20× TaqMan MicroRNA Assay reagent in a total volume of 20 μL. Real-time reaction was carried out at 95 °C for 10 min, followed by 40 cycles of 95 °C for 15 s and 60 °C for 60 s, using the QuantStudio3™ Real-Time PCR System (Applied Biosystems, Milan, Italy) with automatic baseline settings. All reactions, including the no-template controls, were run in triplicate. The relative expression levels of each miRNA in comparison with the normalizer were calculated using the comparative threshold (Ct) method 2^−ΔCt^ [86]. Expression levels (2^−ΔCt^) were log-transformed to better fit a normal distribution.

### 4.5. Pathway Enrichment Analysis of the Predicted Targets

The MicroRNA ENrichment TURned NETwork (MIENTURNET) web tool (http://userver.bio.uniroma1.it/apps/mienturnet/, accessed on 2 September 2024) [87] was used to access the miRTarBase 9.0 database to search for experimentally validated target genes of the myomiRs analyzed in our study and to generate a graphical representation of the network of miRNA–target interactions identified by the enrichment analysis. Networks were constructed considering miRNA–mRNA interactions with strong functional levels of evidence (such as Western blot and luciferase assay), a threshold of 1 for the minimum number of miR–target interactions, and FDR < 0.05. The REACTOME database was used for pathway enrichment analysis to search pathways related to miRNA targets with strong empirical evidence and FDR < 0.01. The MIENTURNET tool was also used to graph enriched plots, where each dot indicates one miRNA whose targets participate in the corresponding pathway. The dots are colored according to the *p*-adjusted value and sized according to the ratio of genes to pathways.

### 4.6. Statistical Analysis

The descriptive data were presented as means (SD) for continuous variables and percentages for categorical variables. The Shapiro–Wilk test was used to evaluate the distribution of variables. Demographic and clinical variables were compared between groups using an independent sample *t*-test for continuous variables and Fisher’s exact test for categorical variables. Correlation analyses were performed using Spearman’s correlation coefficient to determine the magnitude of association between plasma miRNA levels and biochemical markers. A binary logistic regression analysis was used to evaluate the association between the presence of CVD and the variability of the assessed plasma miRNA levels. Multivariate analysis was performed after adjustment for age, sex, and use of statins to exclude their effect. Receiver operating characteristic (ROC) curve analysis was performed to obtain area under the curve (AUC) values for evaluating diagnostic performance of each plasma miRNA for CVD.

Survival data for all-cause of mortality were available for patients, gathered during an average of three years. For each miRNA, Kaplan–Meier survival curves of subjects in the highest quartile were compared to those of remaining quartiles by using the log-rank test. Cox proportional hazard regression analyses were also performed considering age and gender as confounder variables. *p*  <  0.05 was considered statistically significant. All statistical data were analyzed with R (v4.3.3).

## 5. Conclusions

This study found the downregulation of miR-133a in heart failure and miR-133b in stroke, as well as overall decreased expression of miR-133b and miR-206 in patients with CVD. Although in our cohort these miRNAs showed a poor discriminatory accuracy in identifying people with/without CVD, they provide additional support on the role of myomiRs in the pathogenesis of various cardiovascular diseases. Although in silico functional characterization of their targets and pathways revealed their involvement in critical cardiovascular processes, further research is needed to fully elucidate their mechanisms and clinical utility. The results reported here may provide insights into the potential contribution of myomiRs in the cardiovascular injury framework, also offering indications for new research directions.

## Figures and Tables

**Table 1:** Demographic and clinical characteristics of participants stratified by CVD status.

Variables	CVD− (N = 119)	CVD+ (N = 90)	*p*-Value
Age (mean, SD)	82 (7.8)	85 (6.3)	<0.001
Sex (men, %)	33 (28%)	28 (31%)	0.705
BMI, Kg/m^2^ (mean, SD)	26 (6.1)	25 (6.3)	0.813
SBP, mmHg (mean, SD)	128 (11)	126 (13)	0.353
DBP, mmHg (mean, SD)	75 (7.7)	71 (8.2)	0.010
Total cholesterol, mg/dL (mean, SD)	164 (41)	149 (39)	0.009
HDL cholesterol, mg/dL (mean, SD)	49 (15)	49 (12)	0.958
LDL cholesterol, mg/dL (mean, SD)	92 (32)	78 (32)	0.004
Triglycerides, mg/dL (mean, SD)	120 (69)	118 (64)	0.882
Statin users (Yes, %)	22 (18%)	26 (29%)	0.109
Fasting plasma glucose, mg/dL (mean, SD)	103 (48)	98 (28)	0.333
Glycated hemoglobin A1c, % (mean, SD)	6.2 (1.6)	7 (8.6)	0.385
Total protein, g/dL (mean, SD)	6.5 (0.59)	6.5 (0.64)	0.556
Albumin, g/dL (mean, SD)	5.3 (7.6)	5.3 (6.7)	0.787
C-reactive protein, mg/L (mean, SD)	14 (23)	17 (35)	0.498
Urea, mg/dL (mean, SD)	46 (17)	54 (29)	0.019
Creatinine, mg/dL (mean, SD)	1 (0.3)	1.2 (0.58)	0.029
Uric acid, mg/dL (mean, SD)	4.4 (1.2)	6 (7.5)	0.030
Sodium, mM/L (mean, SD)	141 (2.7)	140 (2.8)	0.016
Potassium, mM/L (mean, SD)	4.4 (0.5)	4.5 (0.64)	0.365
Chloride, mM/L (mean, SD)	105 (3.9)	101 (15)	0.014
Calcium, mg/dL (mean, SD)	9.1 (0.62)	9 (0.65)	0.612
Phosphorus, mg/dL (mean, SD)	3.5 (0.92)	3.5 (0.64)	0.962
Magnesium, mg/dL (mean, SD)	1.9 (0.33)	1.9 (0.34)	0.247
Iron, μg/dL (mean, SD)	56 (26)	49 (24)	0.087
Ferritin, ng/mL (mean, SD)	189 (249)	160 (228)	0.432
Total bilirubin, mg/dL (mean, SD)	0.72 (0.39)	0.64 (0.31)	0.112
Hypertension (Yes, %)	80 (67%)	73 (81%)	0.037
Ischemic cardiomyopathy (Yes, %)	-	47 (52%)	-
Atrial fibrillation (Yes, %)	-	26 (29%)	-
Heart failure (Yes, %)	-	16 (18%)	-
Stroke (Yes, %)	-	28 (31%)	-
Deceased (Yes, %)	28 (27%)	40 (49%)	0.004

Abbreviations: SD, standard deviation; BMI, body mass index.

**Table 2:** Logistic regression analysis of miRNA levels and CVD risk.

Predictors	Odds Ratios	CI	*p*	Odds Ratios	CI	*p*	Odds Ratios	CI	*p*
(Intercept)	0.04	0.00–2.97	0.146	0.07	0.00–5.18	0.226	0.02	0.00–2.48	0.147
Age	1.05	1.00–1.10	0.044	1.04	0.99–1.09	0.100	1.06	1.01–1.11	0.028
Sex	0.97	0.45–2.00	0.932	0.92	0.42–1.94	0.835	0.90	0.41–1.87	0.776
Statin	1.51	0.66–3.76	0.353	1.37	0.59–3.44	0.475	1.60	0.68–4.13	0.301
miR-133a	0.71	0.42–1.14	0.170						
miR-133b				0.66	0.44–0.97	0.038			
miR-206							0.57	0.36–0.85	0.009

**Table 3:** Association analysis of miRNAs with specific CVDs.

	Ischemic Cardiomyopathy		Atrial Fibrillation		Heart Failure		Stroke	
miRNA	OR (95% CI) ^#^	*p* *	OR (95% CI) ^#^	*p* *	OR (95% CI) ^#^	*p* *	OR (95% CI) ^#^	*p* *
miR-133a	1.02 (0.63–1.70)	0.943	1.16 (0.64–2.22)	0.647	0.47 (0.24–0.95)	0.031	1.02 (0.58–1.89)	0.935
miR-133b	0.84 (0.57–1.23)	0.378	0.85 (0.52–1.38)	0.517	0.82 (0.46–1.44)	0.495	0.63 (0.39–0.99)	0.048
miR-206	1.28 (0.89–1.89)	0.202	1.06 (0.68- 1.73)	0.795	0.64 (0.38–1.08)	0.090	0.82 (0.54–1.24)	0.332

* *p*-values are adjusted for age, sex, and statin use; *p* < 0.05 was considered significant. ^#^ OR, odds ratio; CI, confidence interval.

## Data Availability

The data that support the findings of this study are available from the corresponding author upon request.

## Supplementary Materials

The supporting information can be downloaded at: https://www.mdpi.com/article/10.3390/ijms252312772/s1.
