# Supplementary material for: The Potential Contribution of MyomiRs miR-133a-3p, -133b, and -206 Dysregulation in Cardiovascular Disease Risk
Source: Int J Mol Sci. 2024 Nov 27;25(23):12772. doi: 10.3390/ijms252312772 (PMC11641116; doi:10.3390/ijms252312772)
Supplement: Supplementary file 1 [file ijms-25-12772-s001.zip › ijms-3330272-supplementary.pdf]

International Journal of
Molecular Sciences

MDPI

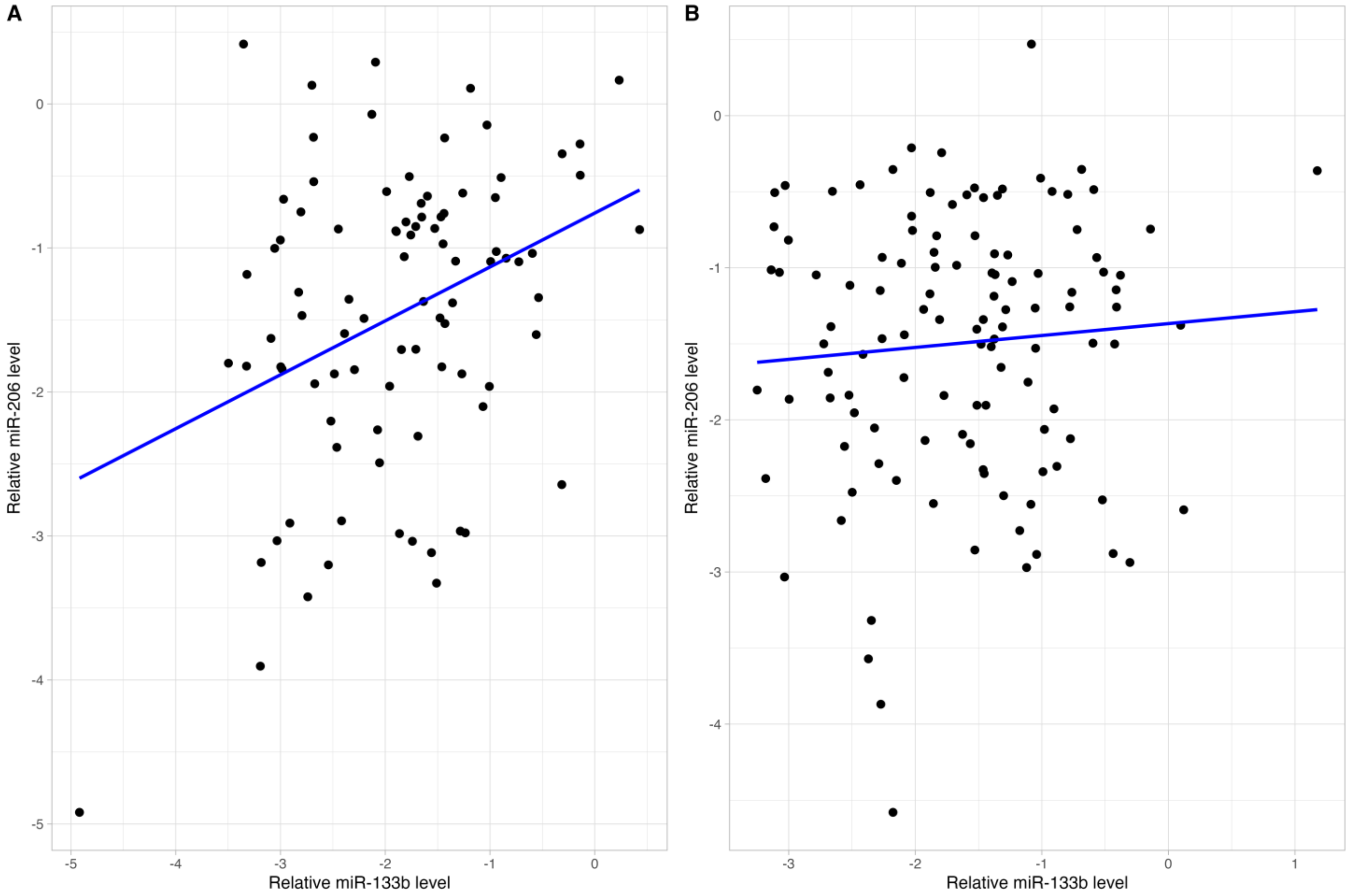

Figure S1. Correlation scatter plot depicting the relationship between the between miR-133b and miR-206 expression levels in (A) the CVD group (r = 0.341; p < 0.001); and (B) the control group (r = 0.076; p >0 .05).

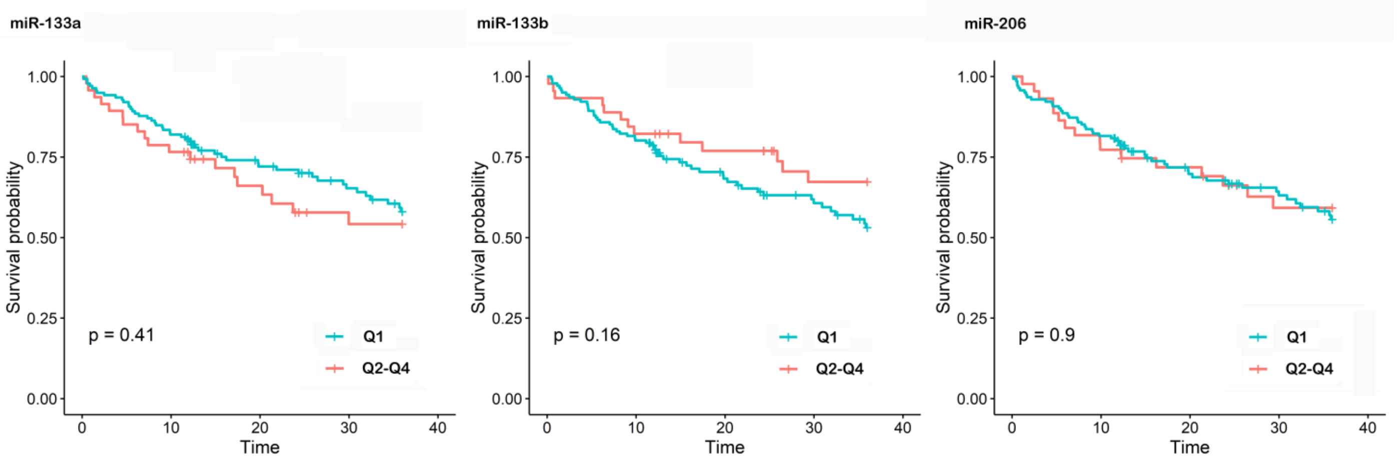

Figure S2. Kaplan-Meier survival curves of the three miRNAs in CVD patients. The p-value refers to the comparison between the highest quartile (Q1) versus the others (Q2–Q4), as assessed using the log-rank test.
